# Supplementary material for: Short-wave magnons with multipole spin precession detected in the topological bands of a skyrmion lattice
Source: Commun Mater. 2025 Jul 4;6(1):139. doi: 10.1038/s43246-025-00858-4 (PMC12227311; doi:10.1038/s43246-025-00858-4)
Supplement: Supplementary file 2 — Supplementary Materials [file 43246_2025_858_MOESM2_ESM.pdf]

Supplementary Materials for
Short-wave magnons with multipole spin precession detected in the topological bands of a skyrmion lattice

Ping Che,[1*†‡] Riccardo Ciola,[2*] Markus Garst,[2,3†]
Volodymyr Kravchuk,[2,4] Priya R. Baral,[5] Arnaud Magrez,[5]
Helmuth Berger,[5] Thomas Schönenberger,[6] Henrik M. Rønnow,[6]
Dirk Grundler,[1,7†]

[1]Laboratory of Nanoscale Magnetic Materials and Magnonics, Institute of Materials (IMX),
École Polytechnique Fédérale de Lausanne (EPFL), 1015 Lausanne, Switzerland
[2]Institut für Theoretische Festkörperphysik, Karlsruhe Institute of Technology,
D-76131 Karlsruhe, Germany
[3]Institute for Quantum Materials and Technology, Karlsruhe Institute of Technology,
D-76131 Karlsruhe, Germany
[4] Bogolyubov Institute for Theoretical Physics of the National Academy of Sciences of Ukraine,
03143 Kyiv, Ukraine
[5]Crystal Growth Facility, Institut de Physique,
École Polytechnique Fédérale de Lausanne (EPFL), 1015 Lausanne, Switzerland
[6]Laboratory for Quantum Magnetism, Institute of Physics,
École Polytechnique Fédérale de Lausanne (EPFL), 1015 Lausanne, Switzerland
[7]Institute of Electrical and Micro Engineering (IEM),
École Polytechnique Fédérale de Lausanne (EPFL), 1015 Lausanne, Switzerland

[*]These authors contributed equally.
[†]To whom correspondence should be addressed:
dirk.grundler@epfl.ch, markus.garst@kit.edu, ping.che@epfl.ch.
[‡]Present address: Laboratoire Albert Fert, CNRS, Thales,
Université Paris-Saclay, Palaiseau 91767, France.

Contents

S1 Temperature-versus-field histories **3**

S2 Phase diagram of the Cu_2OSeO_3 sample **4**

S3 Brillouin light scattering spectra of high temperature skyrmion lattice phase at T = 50 K **6**

S4 Brillouin light scattering spectra attributed to different domain configurations in the laser focus **7**

S5 Meta-stable skyrmion lattice resonance at multiple temperatures **8**

S6 Parameter fit in the field-polarised phase **10**

S7 Dependence of the reciprocal skyrmion lattice vector on the magnetic field **11**

S8 Illustration of magnon modes of the skyrmion lattice with finite wavevectors **12**

S9 Theoretically evaluated BLS intensities for various magnetic fields **13**

S1 Temperature-versus-field histories

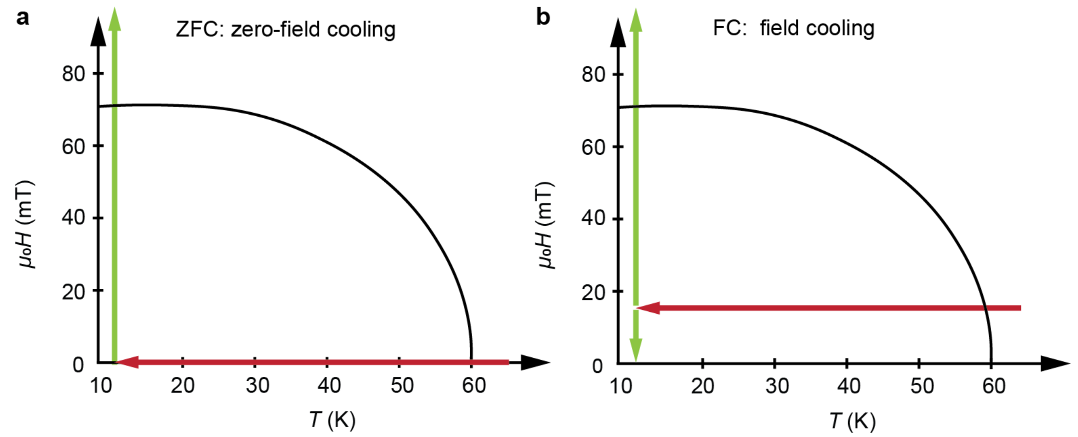

Figure S1: **Sketch of temperature-versus-field histories. a** Zero-field cooling (ZFC) at $\mu_0 H_{\mathrm{FC}}$ = 0 mT. **b** Field cooling (FC) at $\mu_0 H_{FC}$ = 16 mT. Red arrow indicates the cooling field and green arrow indicate the field scanning direction. The cooling process are described in the Appendix section in the main text.

S2 Phase diagram of the Cu_2OSeO_3 sample

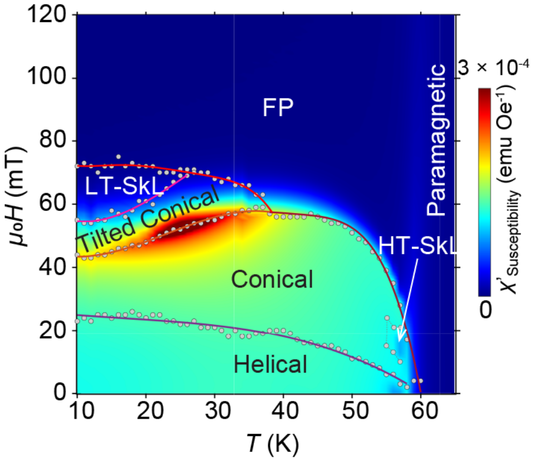

Figure S2: **Phase diagram of the Cu_2OSeO_3 sample.** Phase diagram of Cu_2OSeO_3 obtained by AC susceptibility measurements with the field and temperature history specified in the text below.

The magnetic phase diagram of the Cu_2OSeO_3 single crystal used for the Brillouin light scattering (BLS) experiment was mapped below the transition temperature $T_C \approx 57$ K. The AC Measurement System (ACMS) option of the 14 T Quantum Design Physical Property Measurement System (PPMS) was used. The AC excitation signal was kept at a constant amplitude of 1 Oe and a frequency of 1111 Hz. In order to obtain coherent results, the DC magnetic field was varied along the crystallographic axis used in our BLS setup. Isothermal magnetic field scan-up were performed at 1 K interval from 65 K to 10 K. After the isothermal scan-up at each temperature, a demagnetizing process was performed by applying the slowly oscillating magnetic field from 200 Oe to zero. This process was conducted between each successive isothermal field scan-ups to minimise any hysteresis effects. Phase boundaries were determined using two components of the total susceptibility: $\chi'_{\text{Susceptibility}}$ and $\chi''_{\text{Susceptibility}}$. The high-temperature skyrmion lattice phase (HT-SkL) can be readily identified near T_C as a dip in $\chi'_{\text{Susceptibility}}(T)$. A weak signal corresponding to the helical to conical transition is observed throughout the

phase diagram. As the temperature decreases gradually, non-trivial features begin to emerge in both the $\chi'_{\mathrm{Susceptibility}}$ and the $\chi''_{\mathrm{Susceptibility}}$ components below 40 K, which are consistent with previous reports [1, 2]. Similar to the Ref. [2], an increase in the $\chi''_{\mathrm{Susceptibility}}$ response is observed in the vicinity of the field-polarized state. The features observed in our single crystalline Cu_2OSeO_3 sample can be attributed to the presence of both the tilted conical and low temperature SkL (LT-SkL) phases.

S3 Brillouin light scattering spectra of high temperature skyrmion lattice phase at T = 50 K

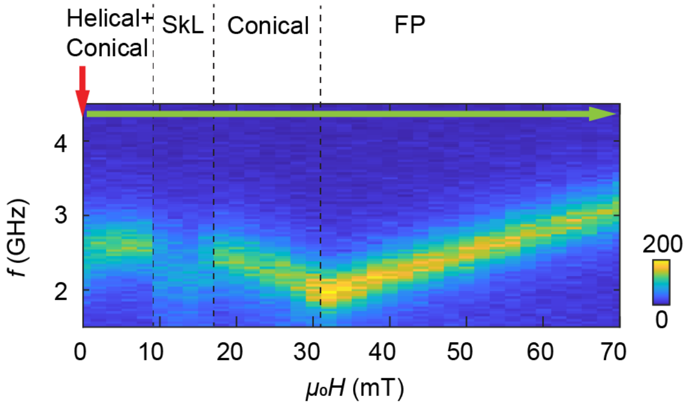

Figure S3: **Brillouin light scattering spectra of zero field cooling at T = 50 K.** Red arrow indicates the cooling field ($\mu_0 H_{FC}$ = 0) and green arrow indicates the field scanning direction. Color bar represents the BLS counts.

BLS spectra were collected at T = 50 K with ZFC process. HT-SkL phase is resolved from 10 mT to 16 mT at T = 50 K. It provides the field range for choosing the cooling field $\mu_0 H_{\mathrm{FC}}$ with FC process to stabilize the meta-stable SkL phases, as discussed in the Appendix section in the main text. There is an discrepancy of 5 K where HT-SkL phase locates in the phase diagram compared with the literature Ref. [3]. This might be caused by the sample fixing on the cold finger inside the cryostate.

S4 Brillouin light scattering spectra attributed to different domain configurations in the laser focus

Here, the two color maps were obtained under the same conditions for the fast cooling process and the BLS measurements. Before every fast cooling process, the sample was warmed up to 100 K to reset the magnetic state. The nucleation of magnetic domains (conical and SkL) might vary in this process thus giving rise to different BLS spectra. In panel **a**, especially above 16 mT, the multipole modes of the SkL resonance discussed in the main text are clearly visible here, indicating a single domain of SkL imaged by BLS. In panel **b**, there is a strong contribution from the conical phase, due to the intensity of the +Q mode whose frequency drops considerably with H, see also Fig. 7 of the main text.

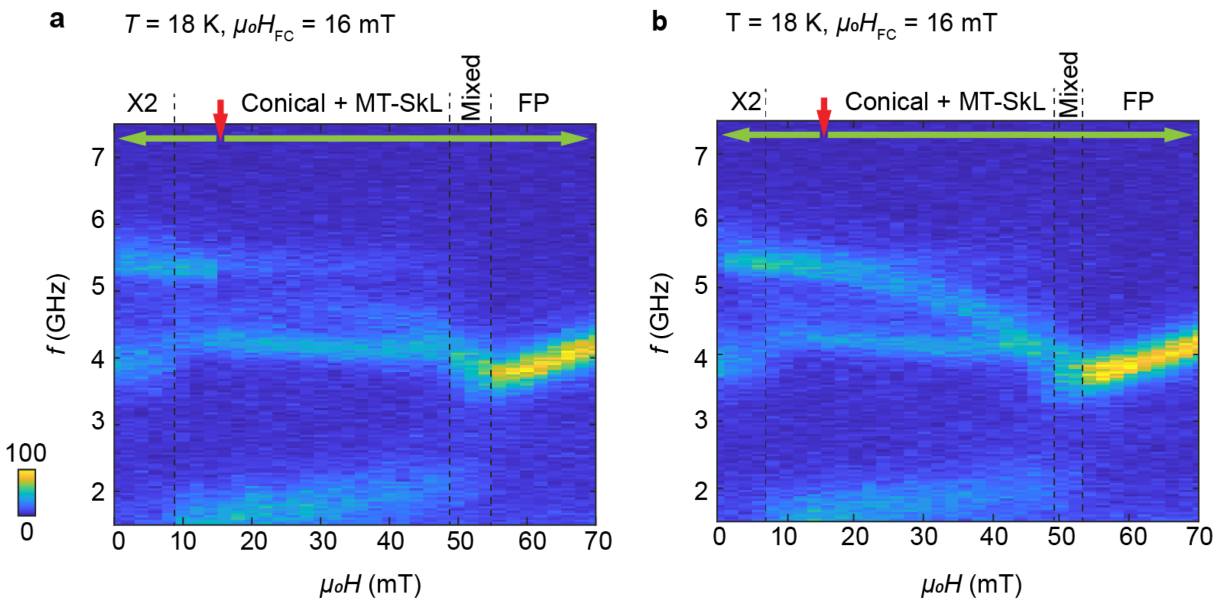

Figure S4: **Brillouin light scattering spectra attributed to different domain configurations in the laser focus.** Anti-Stokes BLS spectra obtained for **H** $\parallel$ [001] at T = 18 K and $\mu_0 H_{\mathrm{FC}}$ = 16 mT via a field cooling process. Color bar represents the BLS counts. Red arrows indicates the $\mu_0 H_{\mathrm{FC}}$ and green arrows show the field scanning directions. The cooling processes of **a** and **b** are operated identically.

S5 Meta-stable skyrmion lattice resonance at multiple temperatures

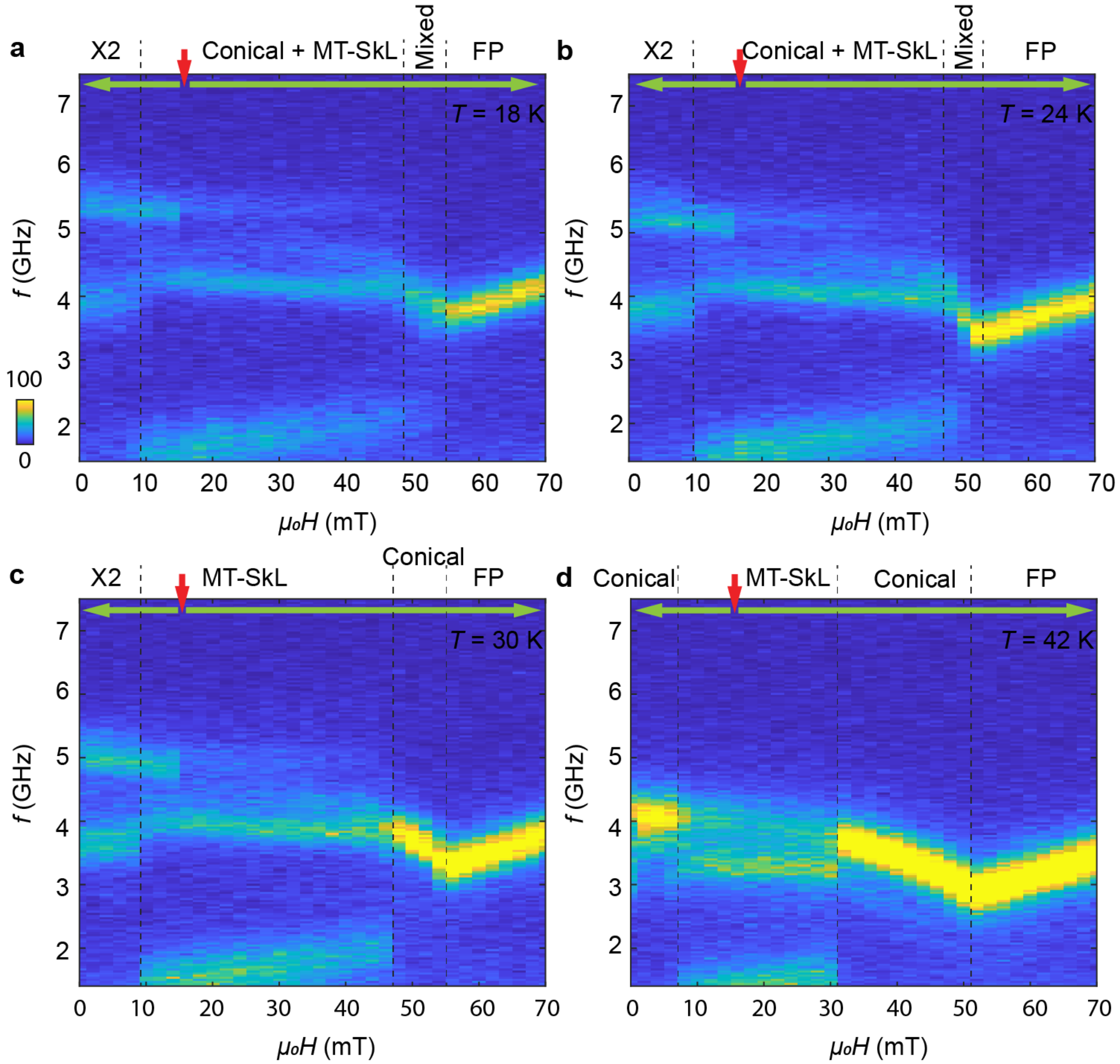

Figure S5: **Brillouin light scattering spectra at multiple temperatures with field cooling.** Anti-Stokes BLS spectra obtained for **H** $\parallel$ [001] via field cooling at $\mu_0 H_{\mathrm{FC}}$ = 16 mT (indicated by the red arrow) and field scan to both directions (green arrows). They are obtained at **a** T = 18 K, **b** T = 24 K, **c** T = 30 K, and **d** T = 42 K. Color bar represents the BLS counts.

Figure S5 shows BLS spectra of the metastable SkL for different temperatures. The extension of the SkL phase across the field axis grows with decreasing temperature. Figure S5 should be compared to the BLS spectra shown in Fig. 4**a** in the main text taken at T = 12 K.

S6 Parameter fit in the field-polarised phase

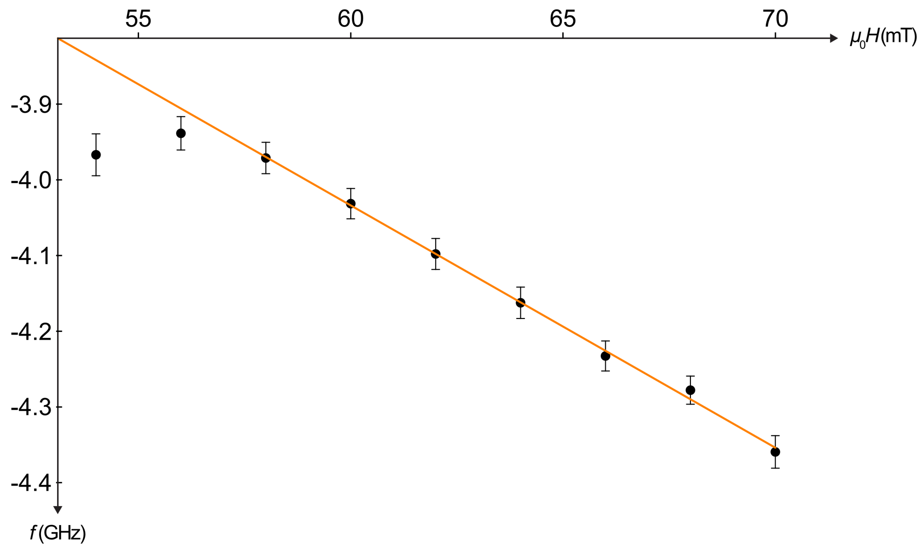

Figure S6: **Linear fit to the experimental measurements in the field-polarised phase.** Experimental frequency positions of the Anti-Stokes resonance in the field-polarised phase (black dots). The theoretical model gives a linear behaviour (orange line) that can be fitted in order to obtain the parameters $\mu_0 H_{c2} = 53$ mT and $\omega_{c2}/2\pi = 2.03$ GHz.

In the field polarised phase it is possible to derive an analytical expression describing the uniform magnon dynamics and its BLS response [4]. Integrating over the micro-focused setup produces a sharp distribution in frequency, see Fig. 7 in the Methods section in the main text.

In the regime of interest, the central peak position is approximately described by a linear relationship between the dimensionless frequency and the dimensionless field

$$\frac{2\pi f_{\text{peak}}}{\omega_{c2}} = A_0 + B_0 \frac{H}{H_{c2}} \,, \tag{S1}$$

with known numerical constants A_0 and B_0, that depend on the geometry of the BLS setup and the sample. Fitting this linear behavior to the experimental data yield the parameters $\mu_0 H_{c2} = 53$ mT and $\omega_{c2}/2\pi = 2.03$ GHz. As discussed in the main text, they do not obey the expected relationship $\omega_{c2} = \gamma_0 \mu_0 H_{c2}^{\text{int}}$ where H_{c2}^{int} is the corresponding internal field.

S7 Dependence of the reciprocal skyrmion lattice vector on the magnetic field

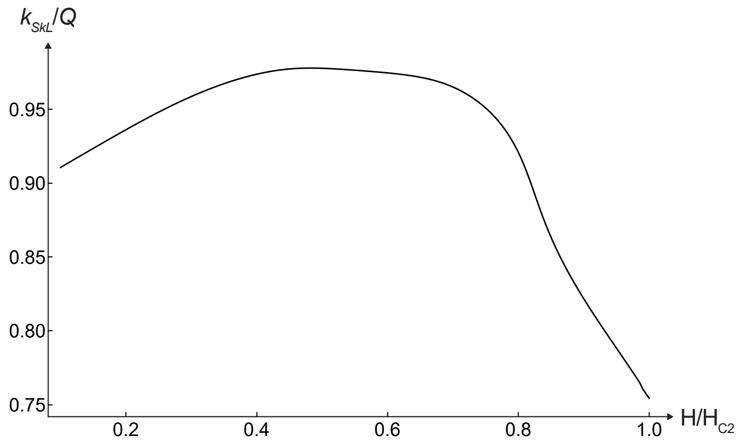

Figure S7: **Size of the reciprocal skyrmion lattice vector k_{SkL} as a function of field.** The theoretically evaluated distance between skyrmions in the skyrmion lattice phase is field dependent resulting in a dependence of the reciprocal lattice vector k_{SkL}; here shown for the parameters of Cu_2OSeO_3 where Q is the helical pitch vector and H_{c2} is the critical field. The field dependence is weak for intermediate fields but it becomes more pronounced for larger fields where the skyrmion lattice phase is also energetically less favorable.

S8 Illustration of magnon modes of the skyrmion lattice with finite wavevectors

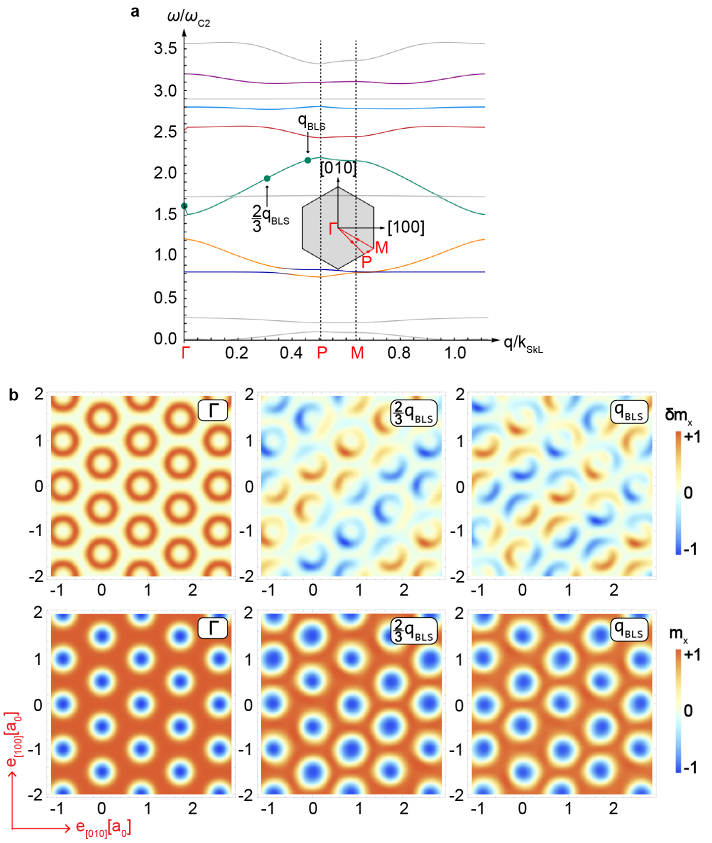

Figure S8: **Illustration of magnon modes of the skyrmion lattice.** **a** Dispersion relation of magnons along the ΓPM-closed-path within the first Brillouin zone at $H = 0.5H_{c2}$ and $q_{||} = 0$. **b** Spatial variation of the breathing mode at a fixed time; its spin wave function $\delta m_x(\mathbf{r}, t = 0)$ (upper row) and the magnetization $m_x(\mathbf{r}, t)$ with an exaggerated spin-wave amplitude (lower row), where $\mathbf{m} = \mathbf{M}/M_s$, are shown for three values of the magnon wavevector along $[1\bar{1}0]$ as indicated in panel **a**. Its time evolution and the one of certain other modes is illustrated in the supplementary videos.

In Fig. 3 of the main text, the time evolution of three modes is illustrated for zero wavevector at the Γ-point of the Brillouin zone. The space and time evolution of counterclockwise (CCW), breathing, clockwise (CW), quadrupole-2 and sextupole-2 modes with finite wavevectors along $[1\bar{1}0]$ is illustrated in Fig. S8 as well as in the five supplementary videos Movie1 to Movie5.

S9 Theoretically evaluated BLS intensities for various magnetic fields

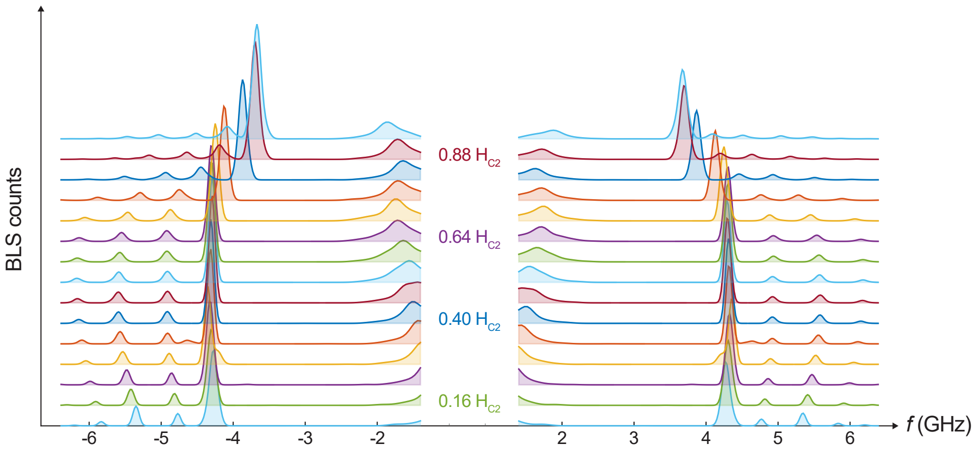

Figure S9: **Theoretically calculated BLS intensity of the skyrmion lattice phase for varying magnetic field.** The negative and positive frequency ranges correspond to the Stokes and anti-Stokes transitions, respectively. The line cuts are separated with respect to each other by a constant offset indicating the increasing magnetic field, from $H = 0.1H_{c2}$ (bottom) up to $H = 0.94H_{c2}$ (top).

Supplementary References

[1] A. Chacon, L. Heinen, M. Halder, A. Bauer, W. Simeth, S. Mühlbauer, H. Berger, M. Garst, A. Rosch, C. Pfleiderer, Observation of two independent skyrmion phases in a chiral magnetic material. *Nat. Phys.* **14**, 936–941 (2018).

[2] M. Halder, A. Chacon, A. Bauer, W. Simeth, S. Mühlbauer, H. Berger, L. Heinen, M. Garst, A. Rosch, C. Pfleiderer, Thermodynamic evidence of a second skyrmion lattice phase and tilted conical phase in Cu_2OSeO_3. *Phys. Rev. B*, **98**, 144429 (2018).

[3] S. Seki, X. Z. Yu, S. Ishiwata, Y. Tokura, Observation of skyrmions in a multiferroic material. *Science* **336**, 198–201 (2012).

[4] N. Ogawa, L. Köhler, M. Garst, S. Toyoda, S. Seki, Y. Tokura, Nonreciprocity of spin waves in the conical helix state. *Proc. Natl. Acad. Sci. U. S. A.* **118**, e2022927118 (2021).
