# Supplementary material for: Short-wave magnons with multipole spin precession detected in the topological bands of a skyrmion lattice
Source: Commun Mater. 2025 Jul 4;6(1):139. doi: 10.1038/s43246-025-00858-4 (PMC12227311; doi:10.1038/s43246-025-00858-4)
Supplement: Supplementary file 3 — Description of Additional Supplementary File [file 43246_2025_858_MOESM3_ESM.pdf]

Description Of Additional Supplementary File

File name: Supplementary Movie 1
Description: Temporal evolution of theoretically calculated spin wave function of the Counterclockwise (CCW) mode

File name: Supplementary Movie 2
Description: Temporal evolution of theoretically calculated spin wave function of the Breathing mode

File name: Supplementary Movie 3
Description: Temporal evolution of theoretically calculated spin wave function of the Clockwise (CW) mode

File name: Supplementary Movie 4
Description: Temporal evolution of theoretically calculated spin wave function of the Quadrupole-2 mode

File name: Supplementary Movie 5
Description: Temporal evolution of theoretically calculated spin wave function of the Sextupole-2 mode.
